# Testing Propositions Derived from Twitter Studies: Generalization and Replication in Computational Social Science

**DOI:** 10.1371/journal.pone.0134270

**Published:** 2015-08-19

**Authors:** Hai Liang, King-wa Fu

**Affiliations:** Journalism and Media Studies Centre, The University of Hong Kong, Hong Kong, China; Hangzhou Normal University, CHINA

## Abstract

Replication is an essential requirement for scientific discovery. The current study aims to generalize and replicate 10 propositions made in previous Twitter studies using a representative dataset. Our findings suggest 6 out of 10 propositions could not be replicated due to the variations of data collection, analytic strategies employed, and inconsistent measurements. The study’s contributions are twofold: First, it systematically summarized and assessed some important claims in the field, which can inform future studies. Second, it proposed a feasible approach to generating a random sample of Twitter users and its associated ego networks, which might serve as a solution for answering social-scientific questions at the individual level without accessing the complete data archive.

## Introduction

Since the increasing popularity of online social media platforms and the rise of computational science, more and more researchers from the natural sciences and engineering have begun to investigate social phenomena using large scale human generated data computationally. This trend facilitates the emergence of computational social science, which aims to use computational approaches to answer questions in the social sciences [1–3].

However, it appears that many of such efforts in computational social science have evolved in isolation from the rest of the discipline of social science. Many findings are largely ignored by mainstream social scientists [3]. In this paper, we argue that two major reasons for the ignorance are lack of generalizability and replicability of the findings. In terms of generalizability, scattered findings in computational social science have not been systematically summarized and generalized for nontechnical social scientists who aim to test social theories. Social scientists are more interested in theorizing social concepts and their relationships across contexts, whereas many scholars in computational studies tend to focus mainly on specific problem solving and algorithmic breakthroughs. Few studies have attempted to summarize or generalize the existing findings [4–6]. As it turns out, the findings have been drawn on a diverse range of measures and study contexts, resulting in unreliable and even contradictory conclusions.

In terms of replicability, there are several sources of bias in current computational social science studies, making the findings hard to replicate [7]. A major obstacle for replication is data representativeness using social media data [8–10]. Many studies are based on non-probability samples. Most of the Twitter study samples are collected by Twitter’s streaming API but this sampling method is nontransparent to researchers and the procedures might be biased toward users making large amounts of posts [9]. Another approach, Breadth-First Search (BFS) crawling, could also be problematic unless all isolated components are collected (see S1 File). The second source of bias may come from differences in measurement of similar concepts. For example, different kinds of networks (e.g., retweeting or reciprocal following networks) are constructed to examine social network properties [5, 6]. Last, online activities on different platforms may represent different types of human behaviors. The platform interface and function can alter people’s behavioral patterns [7].

Given these limitations, we suggest that findings in computational social science using social media data are required to be systematically generalized and replicated. In doing so, we first focus on a single platform, *Twitter*.*com*, to overcome platform variations. Twitter is a combination of social networking services and information sharing applications in which users can make tweets, i.e., posts, with a 140-character limit. Tweets are open online by default, and are also broadcast directly to a user’s followers. Users may rebroadcast a tweet by retweeting (RT) the message to their followers. Alternatively, followers may reply directly to the author. Twitter is one of the most popular social media platforms around the world, and many studies in computational social science are based on Twitter datasets. Therefore, our study scope is limited to only those studies using Twitter data and related to computational social science research.

While accessing the complete Twitter dataset is not possible, our approach is to collect a randomly-selected and representative Twitter dataset. Our method began by generating a list of random Twitter IDs (egos). We then collected all the egos’ alters (i.e., followers and followees) and the following relationships among the alters. Finally, we obtained the profiles and the timelines of the selected users (egos and alters). Although, the sampling approach is not adequate to estimate global network properties, we will show that our dataset is sufficient and possibly a best option to re-examine previous propositions, because results based on random samples could be generalized at the population level, whereas other sampling strategies usually do not have this property (see S1 File). In addition, unlike sampling tweets, sampling users is a more appropriate approach to analyzing individual behaviors.

We synthesize existing studies into three themes to reflect the state-of-art research progress in computational social science in relation to the use of Twitter data, namely usage, network structure, and information diffusion. Although these three themes have covered most findings obtained by observational research, studies using online experiments and combing external data (e.g., survey) for predictions (e.g., voting behavior) are not addressed in this study. We rephrase existing propositions to make them testable at the individual level, assuming that the usage, formation of network structure, and information diffusion can be explained by individual behaviors.

## Materials and Methods

### Ethics Statement

The study was approved by the Human Research Ethics Committee for Non-Clinical Faculties, The University of Hong Kong. Data were obtained from Twitter’s REST API. Before data collection, developer accounts were granted by Twitter to the authors of this study, which allows the access to the data. Indirect identifier data fields will be replaced to unidentifiable pseudo code after all data were collected upon the end of the project.

### Data Collection

Instead of using the streaming API, we used Twitter’s REST APIs to collect a representative Twitter dataset. First, we employed a method reported in Fu and Chan [11] and Zhu et al. [12] to generate random Twitter user IDs. The Twitter ID is a unique (numeric) value that every account on Twitter has. Although an account can change its user name, it can never change its Twitter ID. Therefore, as long as we find an approach to generating a list of valid Twitter user IDs, we find a way to generate a random sample of Twitter users (accounts). After some exploratory experiments, we found that the Twitter ID ranges from 0 to 3,000,000,000 until November 2014. Therefore, we generated 3×30,000 random numbers in this range. And then, we search these numbers via the REST API to check the existence of Twitter IDs. Using this method, we obtained 34,006 valid Twitter user accounts. We call them “egos”. This random sample could represent the population of all Twitter users (see a comparison between the random sampling and BFS sampling strategies in S1 File).

Next, we obtained the egos’ user profiles, alters (followers and followees), and tweets/retweets in user timelines as many as possible. Since users’ tweets and following relationships could be protected by privacy settings, we could only get the public users’ information. For egos, we obtained 4,702,258 tweets from 32,420 egos, of which 15,176 posted nothing. We obtained 2,484,247 unique alters of 32,702 egos, of which 13,713 have zero alters. For alters, we obtained profiles from 2,482,184 users. We further obtained 2,378,687,333 tweets from 1,768,010 alters, of which 124,240 have zero tweet.

Next, we constructed 1.0 ego networks in which nodes are users (egos and alters) and edges are the following relationships (without the following relationships among alters). Users without profiles were excluded. Finally, there are 2,516,190 nodes (including 8,472 ego users) and 3,949,275 edges in the 1.0 ego networks. That means there are 8,472 separated ego networks since only 8,472 egos satisfy the condition that ego users should have at least one alter user and this user’s profile information is publicly available. Among the 8,472 egos, 6,415 users have posted at least one tweet in the past 6 months (active egos). We further obtained the following relationships among the alters of the active egos to construct 1.5 ego networks. We used the 1.5 ego networks to calculate clustering coefficient and betweenness of the active egos. A flow chart of data collection is appended in S1 (Figure A in S1 File).

### Data Analysis

We used a conceptual replication approach. It means that (1) we do not merely reproduce former findings but replicate former conceptual claims using an independent data, and (2) we generalize and rephrase former claims to hypotheses and propositions that can be tested at the individual level. In this way, all analyses in the current study were based on the random sample of ego users. Therefore, findings could be further generalized to the population of Twitter users. Even though we also collected the 1.5 ego networks, we used them to calculate the egos’ network properties, which served as egos’ attributes in formal analyses. The induced alters could not be considered as a representative sample (see S1 File). Further details of the calculations could be found in Table A in S1 File.

## Results

### Usage

#### 20%-80% rule of content generation

The 20/80 rule originally referred to the observation that 80% of Italy’s wealth belonged to only 20% of the population [13]. This rule has been largely believed to be applicable to online peer production systems [14]: Few active users created most of the posts online. Although no clear evidence indicates that the ratio is exactly 20/80, previous studies suggest that the distributions of online production follow power law with exponents ranging from 1.98 to 2.46 on popular social media platforms [15]. In particular, the distribution-follows a power law with an exponent about 1.92 on Twitter [16].

**Fig 1:**
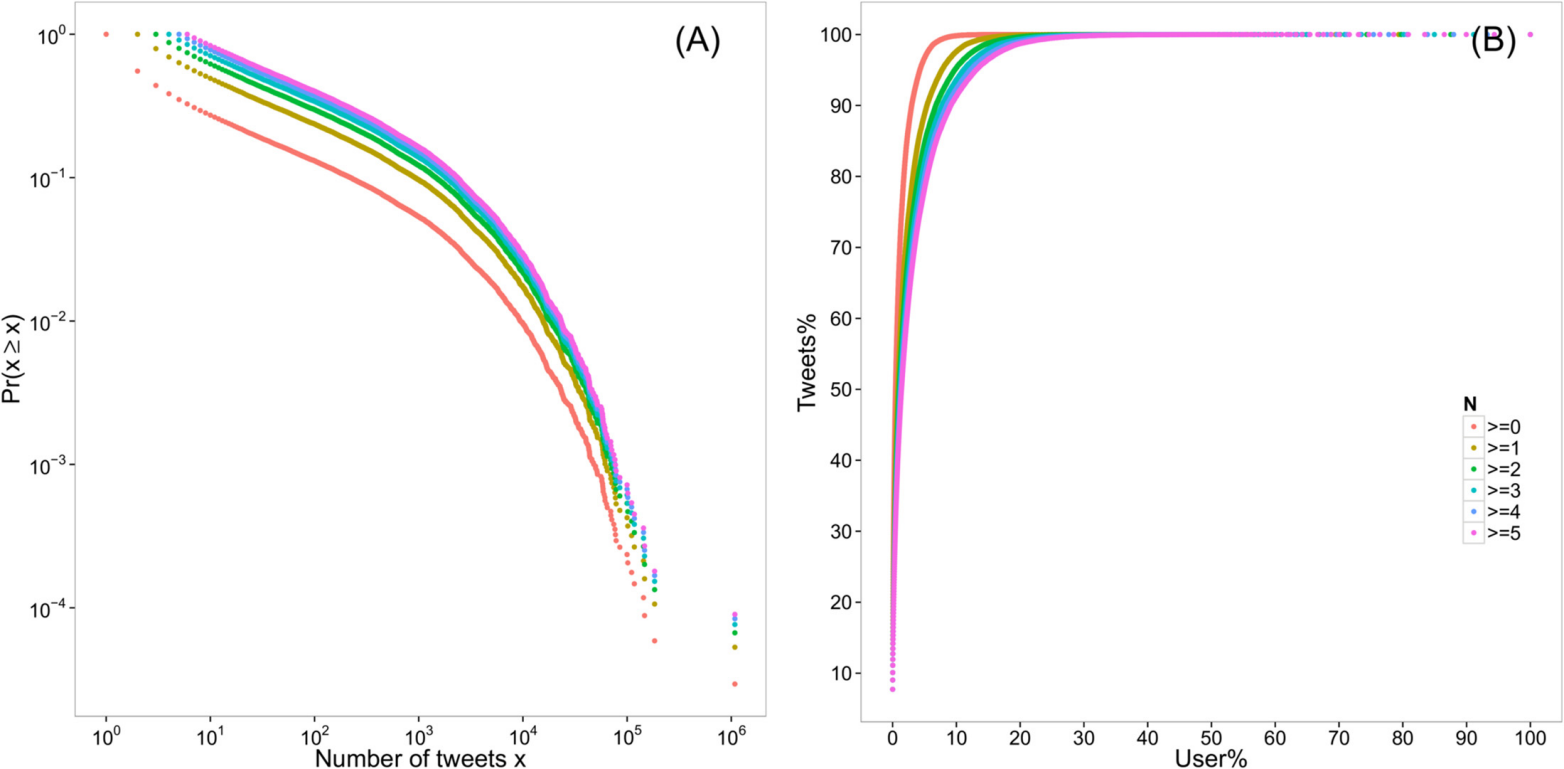
Unequal content generation. (A) Log-log plot of the complementary cumulative distribution functions of the number of tweets per user. (B) Cumulative percentage of tweets created by cumulative percentage of users. Colors indicate that only the users who have posted more than *N* tweets are included. The selection of *N* does not influence the distribution qualitatively. The number of tweets (including original posts, retweets, and replies) for each ego were obtained from the user profile API, and therefore, that is not subject to the 3,200-limit of the user timeline API.

We cannot replicate this power-law hypothesis. Fig 1A shows that the distribution is much flatter than what existing studies have found. We try to fit the distribution using the Clauset-Shalizi-Newman method [17]. The exponent is 1.37 (*min* = 2, *KS* = 0.037, *p* = 0), indicating that the distribution is significantly different from a power law. Yet, the general idea still holds. Fig 1B presents that 2% of users created 80% of tweets, or 20% created nearly 100% of posts. More than half of users didn’t post any tweets at all. The distribution is much more unequal than we expected, and the distribution does not vary across active and less active users.

#### Originality, sociability, and syntactic use

Several features characterizing how users post tweets are frequently mentioned in previous studies—retweet, @-mention, hashtag, and URL. These characteristics are related to the study of originality, sociability, and syntactic use of microblogging. First, Twitter has been celebrated for providing larger amounts of original messages than other platforms. There are fewer duplicates in Twitter than in the Chinese social media platform *Sina Weibo*. The proportion of retweets in Twitter trending topics is 31% [18]. For general topics, the percentage was estimated to be even smaller (3%) [19]. In our sample, the percentage is 22.4% (see point 3.2 in S1 File).

The reply-to and @-mention functions in Twitter have been considered to be indicators for social interactions [20]. However, the estimated proportion of these functions varied drastically, ranging from 22.7% to 86% for @-mention, and from 17.4% to 31% for reply-to [19, 20]. In our sample, 24.1% tweets are replies. There are 49.1% tweets containing @-mention. Note that @-mention could be caused by retweet or reply-to. Excluding these, the proportion of @-mention is 6.9%.

Overall, our findings suggest that there is a moderate level of originality (excluding retweets and replies: 53.5%). More importantly, Twitter platform is more likely to be an interactional platform (Reply+@: 24.1%+6.9% = 31.0%) than an information sharing website (RT: 22.4%).

In terms of syntactic use, previous research found that 20.0% tweets contain hashtags and 29.1% tweets contain URLs [21]. Our results suggest that Twitter users are less likely to use hashtag and URLs than what had been previously reported. Only 14.5% of tweets contain hashtags and 16.5% contain URLs in tweets. These proportions are even smaller in original tweets. According to our data, the percentages are 8.0% and 12.4% respectively. At the user level, more than half of the users have never used hashtags or URLs in their timelines.

#### Circadian rhythms

**Fig 2:**
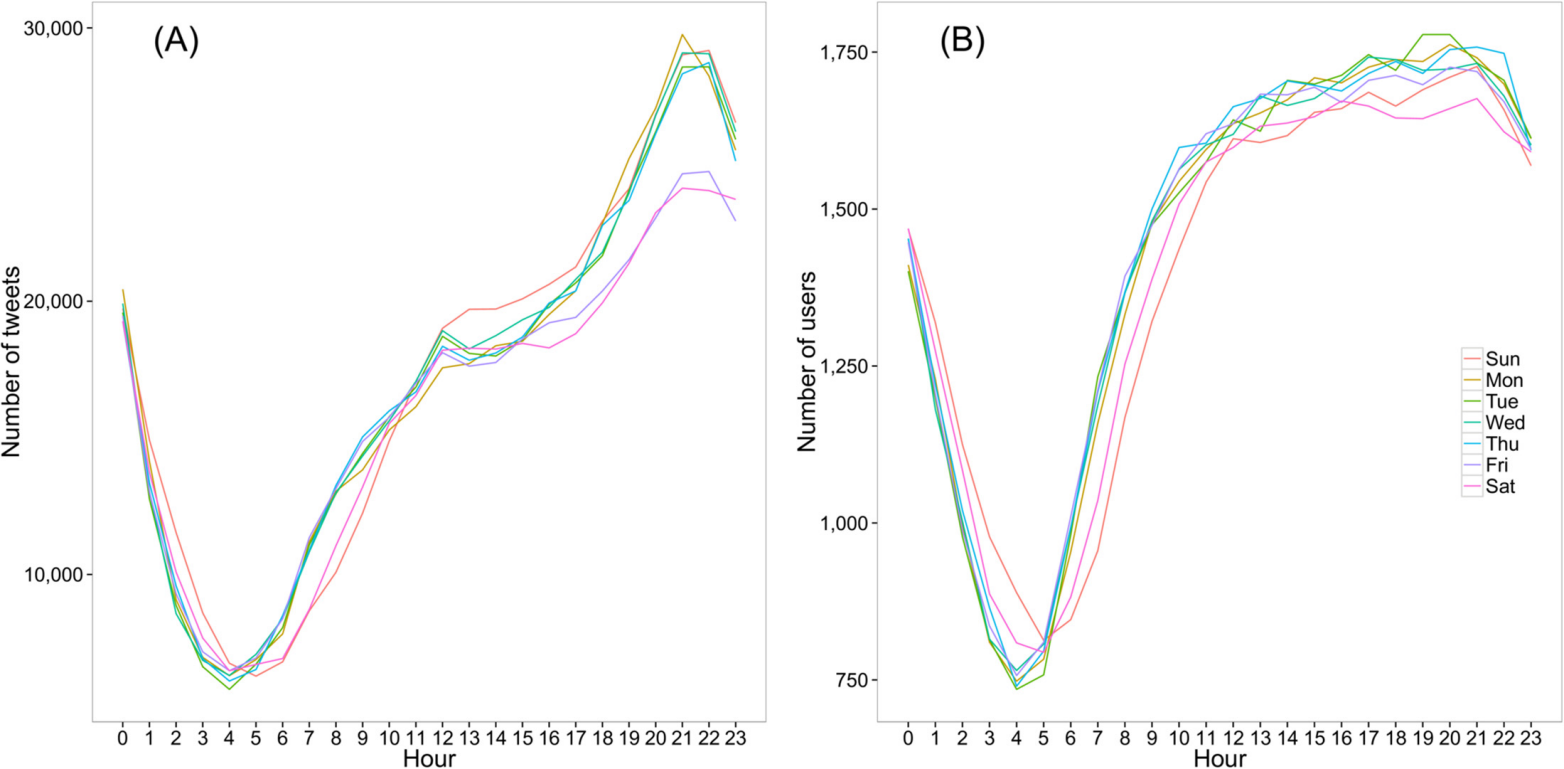
Daily and weekly rhythms of Twitter activity. (A) Tweets posted by hour and day of the week. (B) Number of active users by hour and day of the week. We used the UTC-offset information provided by the REST API to normalize time stamps to local time (see S1 File).

Twitter usage follows the circadian rhythm of the day and week [22]. Researchers found that Twitter messaging activity rises in the morning and increases throughout the day until the evening. Furthermore, they found that weekend use shows a much lower activity and less distinct time of day patterns. These patterns are consistent with the findings in another study based on Facebook data [23]. Our findings are similar to these previous studies. Fig 2 shows that both tweets posting and the number of active users reach their peak around 8:00 p.m., while posting increases more quickly than users during the day time. That means the number of active users remained relatively constant while tweets posted per user increased throughout the day. Besides, users are more active (posting more tweets on average) in workdays than on weekends (*t*_paired_ = -39.43, *df* = 4, 221, *p* < 0.001).

#### Attention and productivity

**Fig 3:**
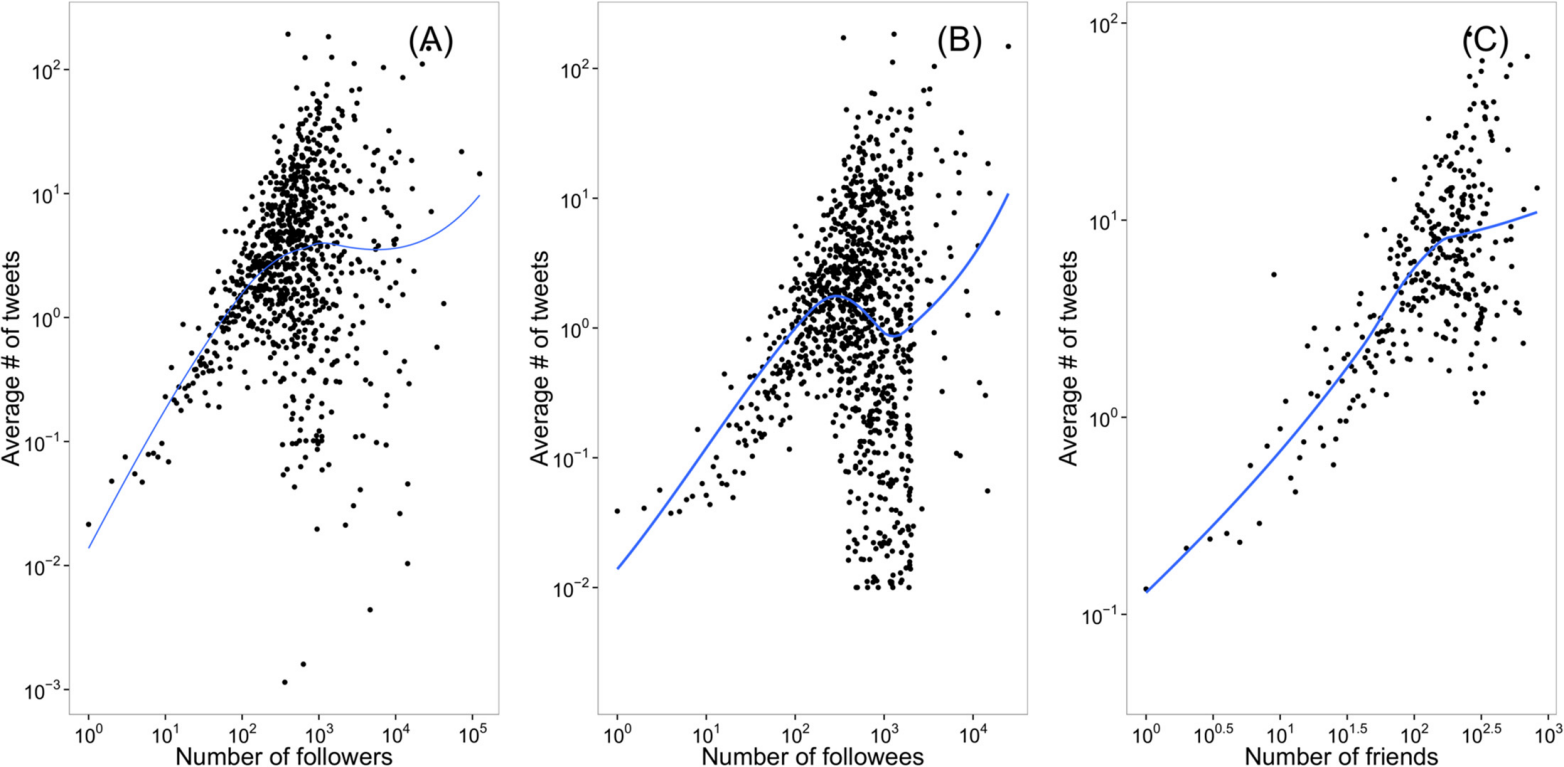
Content productivity and attention received. The average number of tweets as a function of (A) the number of followers, (B) the number of followees, and (C) the number of friends. Friend here is defined as a user who has been mentioned at least twice in an ego’s timeline.

Previous studies suggest that social media users’ productivity exhibits a strong positive association with others’ attention in online social platforms like YouTube [24] and Twitter [14]. Twitter users who receive attention from more people will post more frequently than those who receive less attention. Huberman et al. found that the total number of posts increases with both the number of followers and mentioned friends [14]. However, the number of total posts eventually saturates as a function of the number of followers. Kwak et al. found similar results to Huberman et al. in general. Yet, they found that there are saturation points both for followers and followees [5]. Our findings are consistent with the previous studies as shown in Fig 3. The Pearson correlation between number of followers and the average number of tweets is 0.70 (*t* = 181.86, *df* = 34,004, *p* < 0.001), while the correlation between number of followees and the average number of tweets is 0.55 (*t* = 121.24, *df* = 34,004, *p* < 0.001), and the correlation between number of mentioned users and the average number of tweets is 0.77 (*t* = 224.38, *df* = 34, 004, *p* < 0.001) (S1 File).

### Structure

#### Power-law distribution in follower-followee network

Many studies have sought to examine the social network characteristics of Twitter data by comparing the structural properties to well-known social networks [5, 6]. Although most real world social networks have a power law exponent between 2 and 3, previous studies found very inconsistent results on Twitter follower-followee network [5, 6]. In general, both the number of followee (out-degree) and the number of follower (in-degree) are unequally distributed with heavy tails, resembling power-law distributions. Even though some studies confirmed that power-law exponents are between 2 and 3, others found that the follower or followee distribution is not fit by power law, or their power-law exponents are less than 2 if the distributions are power law [5, 6, 16, 25].

**Fig 4:**
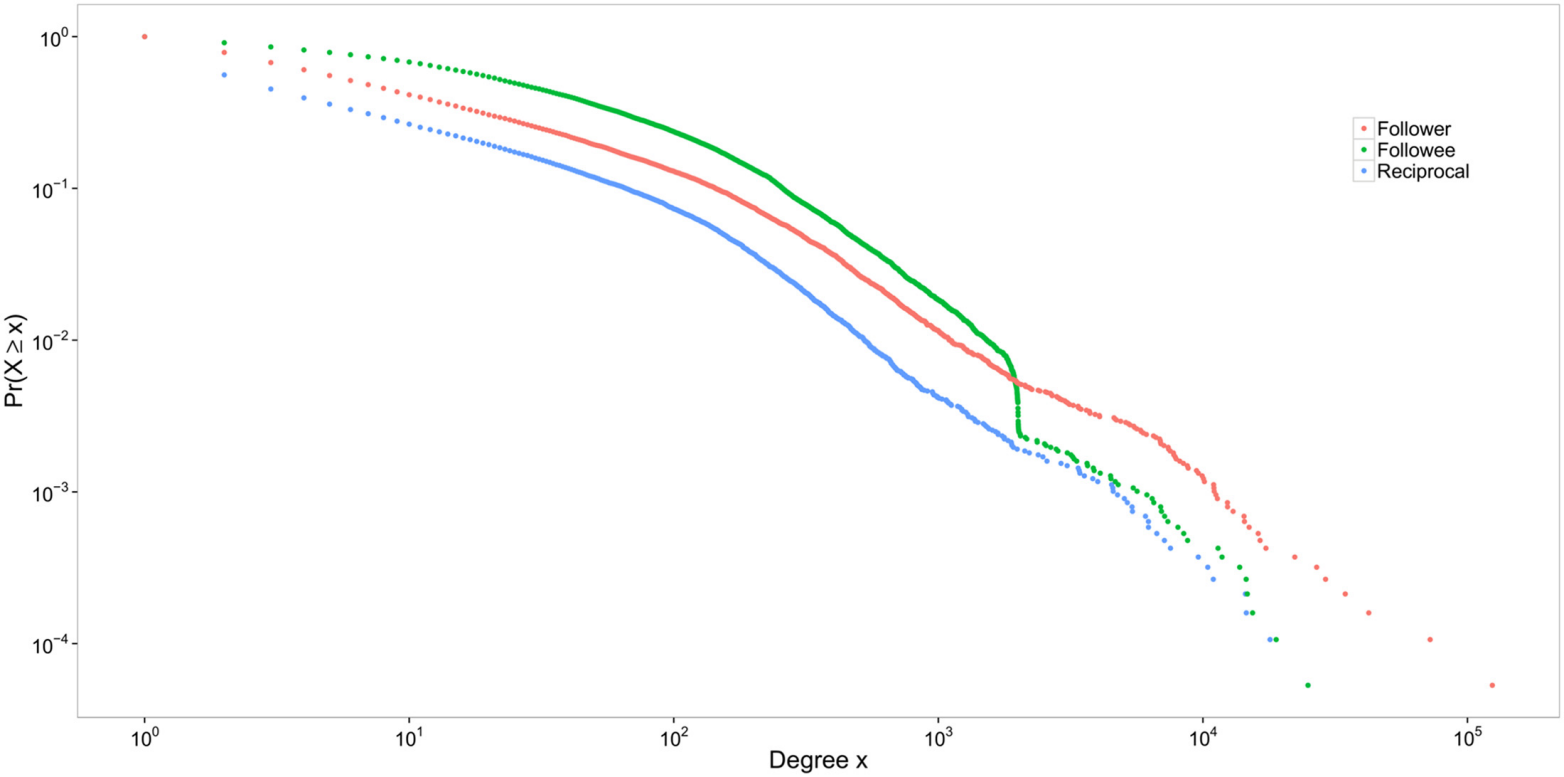
Degree distribution in the follower-followee network. Log-log plot of the complementary cumulative distribution functions of the number of followers, the number of followees, and the number of reciprocal friends. The number of followers and followees for each user were obtained from the user profile API, therefore, are not constrained by the privacy setting for obtaining following relationships.

Using our representative sample, we constructed the three distributions—follower, followee, and reciprocal in Fig 4. Neither follower nor followee distributions are fit by power-law function. The exponent for follower distribution is 1.53 (*min* = 1, *KS* = 0.0185, *p* = 0), while the exponent for followee distribution is 2.32 (*min* = 223, *KS* = 0.0356, *p* = 0). However, the reciprocal degree distribution is fit by power-law with an exponent 2.32 (*min* = 180, *KS* = 0.0213, *p* = 0.332). Overall, it suggests that the follower-followee network does not exhibit the same power-law characteristics as other social networks, whereas the reciprocal friendship does.

#### Formation of the follower-followee network

In addition to the power-law distribution of following networks, several network properties were frequently calculated to gauge the formation mechanisms of the follower-followee network of Twitter. Three mechanisms have been considered to be important: transitivity [26], reciprocity [26], and homophily [27]. The first two are purely structural while the last is based on user attributes.

Transitivity indicates the formation of social ties to people who are friends of existing friends. It can be measured by the clustering coefficient [28]. A former study reported that the average local clustering coefficient is 0.23 for degree = 5 and is 0.19 for degree = 20 in the mutually follower-followee network for active Twitter users [6]. In our random sample, the average clustering coefficient is 0.15 for all active Twitter users in their follower-followee network, while the median is 0.10. Similar to previous findings, the clustering coefficient decreases with increasing degree. The coefficient is 0.29 for degree = 5 and is 0.19 for degree = 20. In the mutual graph, these parameters are slightly lower. The average local clustering coefficient is 0.12 (*mdn* = 0.07) for active users with at least two reciprocal ties.

Reciprocity indicates the relationship that A follows B and then B will follow A back. An earlier study in 2010 found that Twitter shows a low level of reciprocity—22.1%, compared with other social networking sites [5]. In 2014, another study found a much higher reciprocity—42% among active users [6]. Since both studies claim they used Twitter population-scale datasets, it appears that Twitter might evolve to behave more like a social network [6]. However, it can be an outcome of the artifact of selection criteria for active users. In some issue networks (collected by hashtags), the reciprocal ties could be even higher (28%) [16]. Our result is in a number between the two previous findings, 38.3%, which actually reflects the average level of general Twitter users.

Homophily is a tendency that a contact between similar people occurs at a higher rate than among dissimilar people. Many attributes were used to measure user similarity. For instance, users are more likely to follow other users (reciprocally) within closer time zones [5]. For those with ≤ 50 reciprocal friends, the mean time difference is about 1 hour. And for those with ≤ 2,000 reciprocal friends, the median time difference stays below 3 hours. We find a similar result that 50% following relationships occurred between users within one hour time difference.

Another kind of homophily is calculated in terms of user’s popularity (i.e., the number of followers and the number of followees). It means a user is likely to follow other users with similar popularity and they reciprocate. Sometimes, it is also referred to assortativity [29]. On Twitter, the number of followers of a user has been found to be positively correlated with the number of followers of his reciprocal friends, indicating a homophily tendency [5]. However, a negative relationship (-0.30) has been reported for all ties no matter if reciprocal or not [6]. In our representative sample, they are less inclined to follow the users with similar number of followers. The Spearman correlation is -0.31 (*S* = 1.35^19^, *p* < 0.001, *N* = 3,949,275). Nevertheless, the reciprocal ties are more inclined to occur between users with similar degree popularity. The correlation is 0.25 (*S* = 5.40^16^, *p* < 0.001, *N* = 756,445), which is a typical value for social networks [6].

In addition to geo-location and degree similarity, other homophonous attributes have been well documented, e.g., topic interest [30], political alignment [31], happiness [32], well-being [33], and language [34]. We found that 75% of ties were connected between the users using a same language.

#### Dunbar’s number

Forming social ties is subject to cognitive or biological constraints of the brain regarding social interaction. The limit for humans’ social network size is about 150 individuals [35]. Replication studies on social platforms suggest that online interaction is still subject to this constraint [20, 36, 37]. Particularly, users can entertain a maximum of 100–200 stable relationships (reply-to) on Twitter [20].

**Fig 5:**
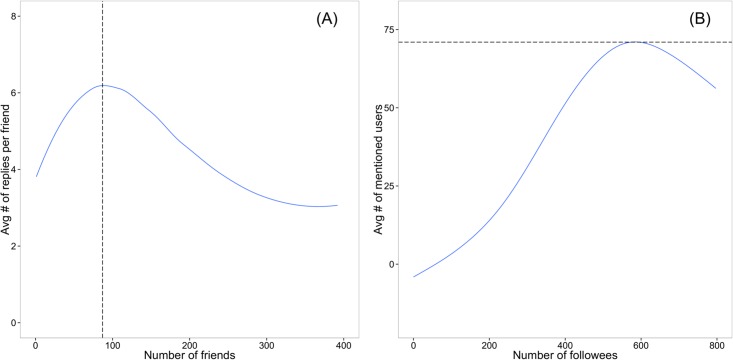
Measuring Dunbar’s number. (A) The average number of replies made by users with different number of friends. (B) The average number of mentioned users as a function of number of followees.

The Dunbar’s number on Twitter was estimated based on the average number of replies sent by the users to their friends [20]. Friend is operationalized as an alter to whom the ego has sent at least one reply. The average number of replies per friend as a function of the number of friends reaches its maximum at around 80 friends, indicating the effect of the cognitive limits of human brain on the ability to maintain social relationships. Fig 5A shows that this maximum in our random sample is approximately 87.

Another measure is based on @-mention [38]. The number of mentioned friends initially increases as the number of followees increases, after a while the number of friends saturates at around 35–40. Fig 5B shows that this number is 71 in our sample, which is much larger than that in the original study.

Although, our results generally supported Dunbar’s social brain hypothesis on Twitter, the estimate of the accurate number varies across measurements and is different from previous findings. We found that different estimations might be simply caused by different time periods of data scraping (Figure C in S1 File).

### Information Diffusion

#### Influential hypothesis

Modeling information diffusion and detecting networking influence on Twitter can be analyzed at both user level and tweet level. At the user level, the dominant framework is the influential hypothesis, which states that a minority of users are more influential than others in terms of triggering retweets. Previous studies suggest a lack of reliable predictors for retweetability (retweeted or not) at the user level. The ranking of the most influential users differed depending on the measures, like the number of followers, PageRank, number of mentions, and retweets count [5, 39]. Nevertheless, few studies found that retweetability is positively associated with the number of followers [40, 41], number of followees [41], the age of the account, and those users belonging to several groups who act as brokers [42].

The variance of retweeting probability comes from both within and between individual users. Therefore, we employed multilevel generalized models for predicting retweetability (being retweeted or not) and retweet count received by the original tweets (see SI). Table 1 contains both user-level and tweet-level factors. For user-level factors, as expected, the number of followers and the age of the user account are positively associated with retweetability. However, the number of followees is negatively associated with retweetability. In addition, clustering coefficient, which indicates the followees and followers of a user are well connected, is positively associated with retweetability. To predict retweet count, we observed several differences with previous studies. Both age of the user and the number of reciprocal ties are negatively correlated with retweet count, whereas the number of followees is positively associated with retweet count.

**Table 1:** Multilevel generalized models predicting retweetability and retweet count.

	Being retweeted or not		Retweet count ( > 0)	
	Estimate (SE)	Z	Estimate (SE)	Z
Log # of followers	0.628 (0.037)	16.77	0.269 (0.015)	18.23
Log # of followees	-0.444 (0.043)	-10.37	0.095 (0.018)	5.22
Log days since created	0.250 (0.005)	49.77	-0.010 (0.001)	-59.36
Log # of reciprocal ties	0.304 (0.052)	5.91	-0.191 (0.021)	-9.05
Clustering coefficient	2.773 (0.316)	8.78	0.645 (0.158)	4.08
Betweenness	-0.342 (0.234)	-1.46	-0.064 (0.093)	-0.69
Presence of hashtags	0.354 (0.008)	44.31	0.143 (0.004)	32.73
Presence of mentions	0.692 (0.008)	85.64	-0.095 (0.005)	-114.00
Presence of URLs	-0.631 (0.010)	-63.78	-0.584 (0.005)	-19.31
Intercept	-6.575 (0.202)	-32.50	-0.065 (0.096)	-0.68
*Variance of intercepts*	2.613		0.236	
*Log-Likelihood*	-499,941.5		-632,406.9	
*Explained variation*	16.5%		3.5%	
*# of users (egos)*	5,894		3,082	
*# of tweets (ego timelines)*	2,039,363		198,199	

#### Source characteristics

At the tweet level, researchers believe that the characteristics of a specific tweet are important for spawning retweets. We call them source characteristics here. The presence of hashtags, URLs, and @-mentions are the most frequently-mentioned predictors. The presence of both hashtag and URL is positively correlated with retweetability [41]. According to Table 1, the presence of hashtags and mentions is positively associated with retweetability. Unexpectedly, the presence of URLs is negatively related to retweetability. A bivariate analysis reveals that the presence of URLs indeed increased retweetability from 10% to 15%. However, URLs always co-occurred with hashtags. That means the correlation between the presence of URLs and retweetability is spurious and induced by the correlation between the presence of hashtags and retweetability. In terms of retweet count, the presence of mentions is negatively correlated, although the effect sizes are the largest in both models.

#### Exposure hypothesis

Unlike above-mentioned studies, which emphasize the probability of being retweeted by other users, the exposure hypothesis focuses on the probability of retweeting other users’ tweets. The hypothesis posits that repeated exposures to an idea are particularly crucial for adopting the idea [43, 44]. On Twitter, successive exposures indeed increase the probability that the user will begin mentioning specific hashtags [45] or URLs [46], though the marginal effect might soon reach its maximum aggregately. Further studies found that the relationship could vary across topics [45], across number of friends due to the information overload of highly connected users [46, 47], and across community structures [48].

**Fig 6:**
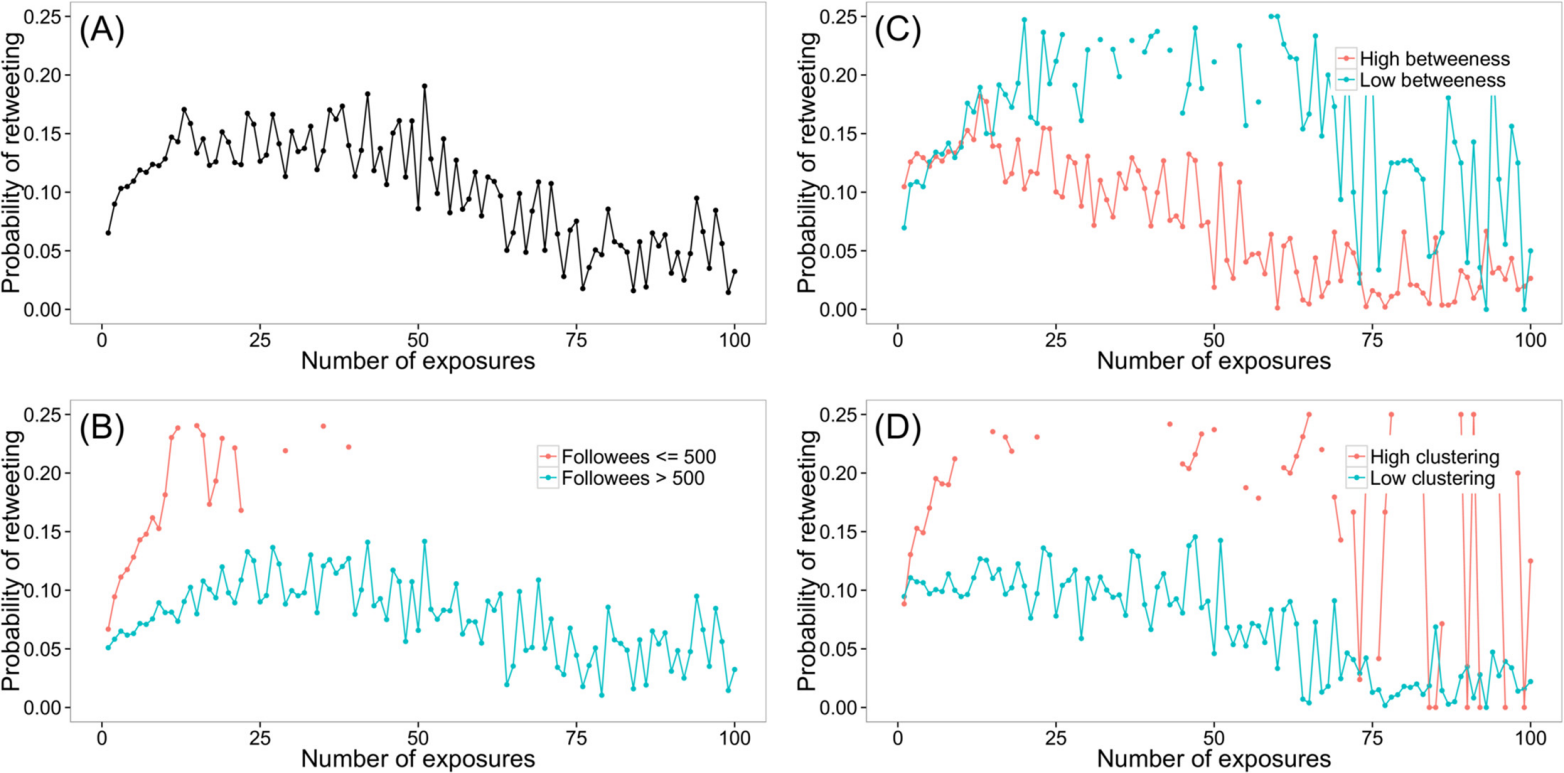
Exposure hypothesis and its variations. The probability of retweeting as a function of the number of followees who have tweeted a post, (A) averaged over all users, (B) by breaking down users into classes based on the number of following friends they have, (C) by breaking down users into classes based on the betweenness in their ego networks, and (D) by breaking down users into classes based on the clustering coefficient in their ego networks. Medians of betweenness and clustering coefficient are used as cut-points for grouping users.

We replicated this hypothesis using the official retweet based on our random sample. Consistent with the former studies, repeated exposures of a tweet indeed increase retweeting probability at the initial stage, but start to decrease around the 20^th^ exposure (Fig 6A). This relationship is stronger for users with fewer followees (Fig 6B), with lower betweenness (Fig 6C), and with higher clustering coefficient (Fig 6D). That means the exposure hypothesis is more likely to be true in small and dense groups.

## Discussion

The current study generalized and replicated 10 propositions related to computational social science using a truly representative Twitter dataset. The study contributes to computational social science studies using social media data in two ways. First, it demonstrated lack of replicability in previous studies. More than half of the propositions could not be fully replicated (Table A in S1). The major reason is believed to be the variation of sampling strategies employed in different studies. For example, the originality and the ratio of using hashtags or URLs in our random sample are lower than those previously found, possibly because former studies merely included active users. The second reason is the variation of methodology of measurements. For example, different methods to estimate Dunbar’s number could result in quite different outcomes. Similarly, the distribution of followers does not follow a power law, however, the distribution of reciprocal degree does. The third reason may come from the variation of analytic strategies. As we mentioned, the presence of URLs is positively associated with retweetability using bivariate analysis. Yet, a multivariate analysis reveals that this correlation is actually spurious. In this sense, it is important for future studies to collect randomly samples and conduct rigor statistical analyses for computational social science research. Second, the study systematically summarized and assessed some important claims in the field, and proposed a feasible approach to generate a random sample of Twitter users and its associated ego networks. On the one hand, the random sampling approach is more appropriate to study social science problems because it satisfies the basic requirement of most statistical models for generalizing claims at the population level. On the other hand, scattered findings were generalized into proportions, which reflects the state-of-art of the field and paves the ways for future studies.

Our replication, to some extent, has corrected some important biases in previous studies using online data. However, more areas should be addressed. First, we only considered a single platform to overcome the design bias. It is unclear whether the confirmed propositions are also correct on other platforms. Future studies should explicitly analyze the impact of platform interfaces. Second, four propositions in our study seem to be robust across sampling strategies. It might be because these propositions reflect the homogeneity of online user behaviors, which means that nearly all users follow similar patterns. Therefore, they appear to be insensitive to sampling strategies. However, future studies need to retest the robustness of these propositions before we can consider them as universal. Third, a growing amount of studies are using experimental design on social media and they are usually supported by the service providers. The uniqueness of this type of study makes the findings hard to replicate. However, the robustness, ethical concern and external validity of social experiments should receive more attention. Finally, there are many studies using online texts to predict voting behaviors [49] and the approval rate of political actors [50]. The implication of the current study for this line of research is that we may focus on representative individual users other than posts.

## Supporting Information

S1 File Information S1 documented 1 table (Table A) and 3 figures (Figure A-C). (PDF)
(PDF)
