# Supplementary material for: Testing Propositions Derived from Twitter Studies: Generalization and Replication in Computational Social Science
Source: PLoS One. 2015 Aug 19;10(8):e0134270. doi: 10.1371/journal.pone.0134270 (PMC4546006; doi:10.1371/journal.pone.0134270)
Supplement: S1 File — (PDF) (PDF) [file pone.0134270.s001.pdf]

Supplementary Information

Testing Propositions Derived from Twitter Studies:
Generalization and Replication in Computational Social Science

Hai Liang, King-wa Fu

1. Data collection flow chart

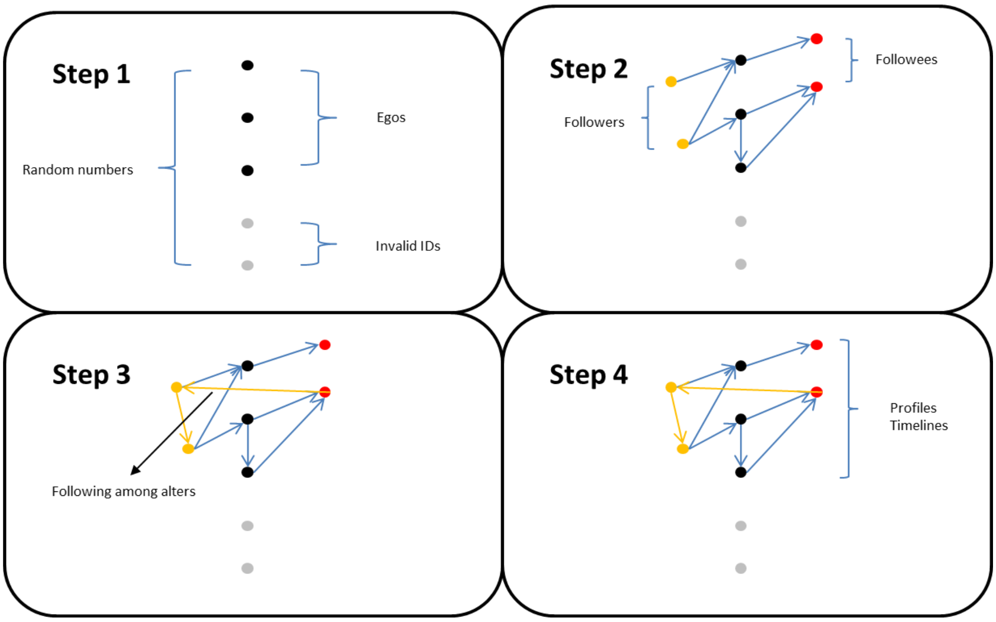

Figure A | An illustration of the data collection procedure. Actually, we collected user profiles and timelines at step 1 and step 2 as soon as we obtained the Twitter IDs. It is equivalent to collect them once at a step (step 4).

2. Validation of representativeness

Given the lack of ground truth about the population of Twitter users, we conducted cross-validations to test the internal validity of our sampling approach. In doing so, we repeated the procedure described in Fig. S1 three times independently. Therefore, we got three independent samples of ego Twitter users, namely ego_batch1 (*N*=11,247), ego_batch2 (*N*=11,631), and ego_batch3 (*N*=11,129). We combined the three batches as a single dataset used in the main text. From the egos, we got three batches of alters respectively, alter_batch1 (*N*=844,533), alter_batch2 (*N*=957,489), and alter_batch3 (*N*=868,270).

Our validation method is to test whether there are any differences among batches of users. If the differences are statistically significant, the sampling approach is certainly not representative. Otherwise, we may have some confidence that the sampling approach is reliable. We compared three attributes of the users: the distribution of the number of statuses, the distribution of the number of followers, and the distribution of the number of followees, since all user profiles contain these items.

First, we compared the distributions for ego batches. We employed the

Kolmogorov-Smirnov test to compare distributions. (1) For the number of statuses, the KS-D metric for ego_batch1 and ego_batch2 is 0.0089 ($p = 0.7516$), for ego_batch1 and ego_batch3 is 0.0093 ($p = 0.7126$), while for ego_batch2 and ego_batch3 is 0.0112 ($p = 0.4734$). (2) For the number of followers, the KS-D metric for ego_batch1 and ego_batch2 is 0.007 ($p = 0.9433$), for ego_batch1 and ego_batch3 is 0.0039 ($p = 1$), while for ego_batch2 and ego_batch3 is 0.007 ($p = 0.9449$). (3) For the number of followees, the KS-D metric for ego_batch1 and ego_batch2 is 0.0085 ($p = 0.8053$), for ego_batch1 and ego_batch3 is 0.0104 ($p = 0.5774$), while for ego_batch2 and ego_batch3 is 0.0149 ($p = 0.1574$). All comparisons suggest that there are no significant differences across batches and attributes, indicating a very high level of internal validity of our sampling approach.

Second, we compared the distributions for alter batches. (1) For the number of statuses, the KS-D metric for alter_batch1 and alter_batch2 is 0.0514 ($p < 0.001$), for alter_batch1 and alter_batch3 is 0.046 ($p < 0.001$), while for alter_batch2 and alter_batch3 is 0.0885 ($p < 0.001$). (2) For the number of followers, the KS-D metric for alter_batch1 and alter_batch2 is 0.0902 ($p < 0.001$), for alter_batch1 and alter_batch3 is 0.0551 ($p < 0.001$), while for alter_batch2 and alter_batch3 is 0.1209 ($p < 0.001$). (3) For the number of followees, the KS-D metric for alter_batch1 and alter_batch2 is 0.032 ($p < 0.001$), for alter_batch1 and alter_batch3 is 0.0352 ($p < 0.001$), while for alter_batch2 and alter_batch3 is 0.0547 ($p < 0.001$). All comparisons suggest that distributions are significant different across batches of alters, indicating that the induced alters from representative egos are not representative at all. It also shows that the commonly used BFS sampling approach could not generate a representative sample.

3. Analysis details

Table A. Datasets used for testing propositions

Propositions	Ego profiles	Alter profiles	Ego-alter relationship	Alter-alter relationship	Ego timelines	Alter timelines	Replicated?
1) 20/80 rule	√						N
2) Originality, sociability, and syntactic					√		N
3) Circadian rhythms					√		Y
4) Attention and productivity	√				√		Y
5) Power-law distribution	√		√				N
6) Network formation		√	√	√			Y
7) Dunbar's number					√		N
8) Influential hypothesis	√		√	√	√		N
9) Source characteristics					√		N
10) Exposure hypothesis	√		√	√	√	√	Y

Notes:

1) The 20/80 rule was tested using the number of statuses in ego profiles. We acknowledge that there might be many fake accounts in our sample. However, it might not affect the distribution too much. We could assume that users posted a few tweets are fake accounts. As Fig.1 presented, the selection of N (number of statuses) does not change the distributions. There are 34,006 users when $N \geq 0$, 18,830 when $N \geq 1$, 14,943 when $N \geq 2$, 13,079 when $N \geq 3$, 11,916 when $N \geq 4$, 11,111 when $N \geq 1$.
2) Retweet could be identified by whether the API returned a retweeted user ID. We did not count the unofficial retweet (e.g., "RT: @username") in our study, because it may introduce additional noise. Also, we emphasized that @ could be a byproduct of retweet and reply-to. We explicitly distinguished the induced @ (by replying to others or retweeting) from the @ in original tweets. Both official RT and @ are provided by the Twitter Timeline API.

   Similarly, reply could be identified by whether the API returned a reply-to user ID. Original tweets are statuses that are not replies or retweets. In our ego tweets sample (4,702,258 tweets produced by 17,244 users), the proportion of replies is 24.1% and the proportion of retweets is 22.4%, therefore, the proportion of original tweets is 53.5% (1-24.1%-22.4%).

   The proportions were calculated at the tweet level. We should note that Twitter timeline API has a 3,200 limit for each user. According to ego profiles, the maximum tweets posted by our sampled egos is 1,082,000. However, we do not think it will influence our results in general, because only 2.5% (873) of egos have posted over this limit.
3) Ego timelines were used to examine circadian rhythms. Among the 17,244 egos who have posted at least one tweet, only 4,222 users contain the information of UTC-offset, which we used to normalize the UTC time stamps to local time. This implementation increases the accuracy of our tests based on the 4,222 users, whereas it could cause the problem of generalizability. We do not know whether these users with UTC-offset information truly represent the other users without such information.
4) Fig. 3A and Fig. 3B could be reproduced by using ego profiles. Profile API provides follower count, followee count, and statuses count for each user. Since our sample of users were registered in different years since 2006, the older users certainly posted more statuses and have more alters than the younger counterparts. We calculated the daily average tweets (# of total statuses/days since created) to control this compounding effect. Since all count variables are right-skewed, the correlation coefficients were calculated after log-transformations. Instead of daily average, if we use the original values of tweet count, the correlation between

number of followers and number of tweets is 0.81 (t=247.15, df=34,004, $p <$ 0.001), while the correlation between the number of followees and number of tweets is 0.68 (t=171.69, df=34,004, $p <$ 0.001). Both are larger than those using average tweets, indicating the existence of compounding effect. Number of friends in Fig. 3C was obtained from the ego timeline. For those egos who posted nothing, friend count was set to 0.

5) Ego profiles contain sufficient information to examine the distributions of the number of followers and followees per ego. We need ego-alter relationships to calculate the number of reciprocal ties per ego. In case fake accounts may influence the degree distributions, we delete those users who have not posted anything. Fig. S2 shows that the results are actually similar.

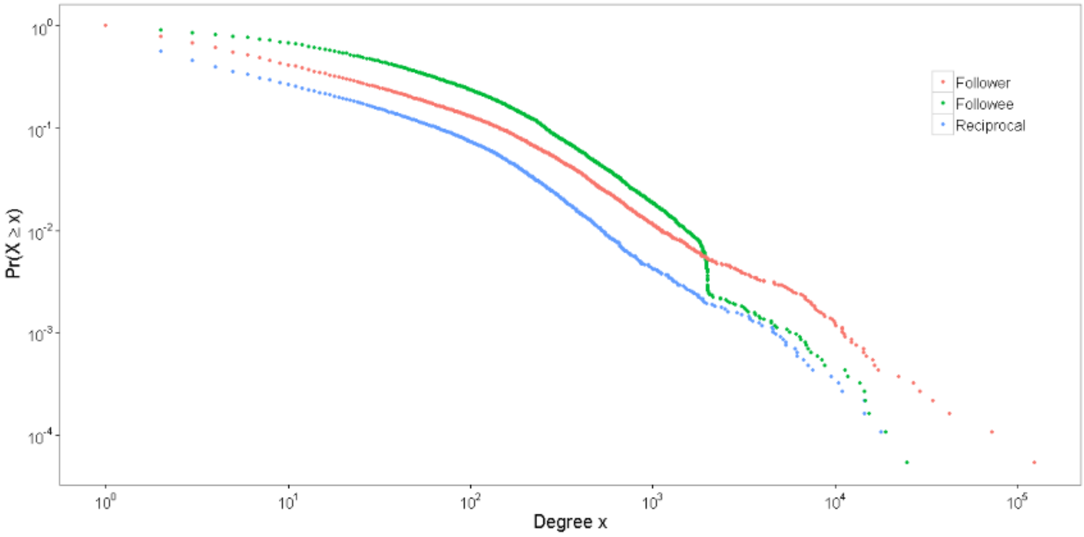

Figure B | Degree distribution in the follower-followee network excluding users with zero post.

6) We calculated the local clustering coefficient for each ego using the ego-alter and alter-alter relationships. In other words, we calculate this coefficient in each 1.5 ego network separately (N = 6,415 active egos). The average clustering coefficient is the mean of the 6,415 local clustering coefficients. Please note that the 1.5 ego network only contains the full triangles of the ego node. Thus, calculating alters' clustering coefficient is meaningless. The mutual graph is the 1.5 ego network excluding non-reciprocal ties and the associated nodes.

7) Fig. 5A shows that the estimated limit is around 87, which is much smaller than 100-200 estimated in Gonçalves et al.[1]. The former study collected the data from the active users in 2009. Active users in different periods may behave quite differently in social interactions. Fig. S3 shows that this cohort effect indeed exists. Users registered before 2009 have a higher limit than their counterparts. It demonstrates that previous studies may only reflect behaviors of a sub-population

of Twitter users, whereas our study reflects the average.

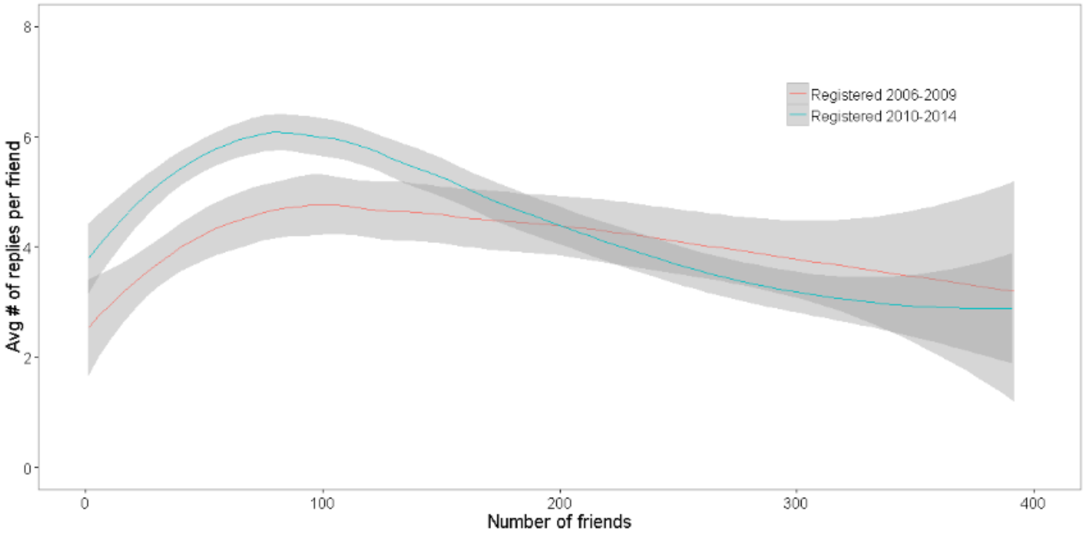

Figure C | Estimating Dunbar's number by breaking down users according to their account registration time.

8) There are two reasons we employed the multilevel generalized linear model[2]. First, the variances of retweetability and retweet count could attribute to the users who posted the tweet (user level) as well as the characteristics of the tweet per se (post level). The user level analysis is related to the influential hypothesis. The post level analysis is related to the source characteristic hypothesis. The multilevel model contains two parts: fixed and random effects. The fixed part estimates the average influences while the random part estimates the variability of the fixed effects across clusters (i.e., users in our study). It could be naïvely (but not actually) understood as that we conducted a separate OLS regression for each ego. The fixed coefficients are the average values of the OLS coefficients. The random coefficients are the variances respectively.

   Second, we used generalized linear model because the dependent variables are binary responses (retweetability) and count data (retweet count). Therefore, the link function for retweetability is the logit (for binomial distribution) while the link function for count is the logarithm (for Poisson distribution). In both models, we only included random intercept effect. Interpretations to the fixed effects are analogue to logistical regression and Poisson regression respectively.

9) Overall, according to the *Z*-scores in Table 1, post level variables are more powerful in predicting retweeting behavior. Another observation is that retweetability is much more predictable than frequency using our variables.

10) The exposure hypothesis focuses on the probability of retweeting alters' tweets by egos. Therefore, we selected the users who has retweeted at least once (7,226 egos). The official RT, rather than hashtag and URL, was used to identify retweet.

This implementation may decrease the proportion of retweets but increase the accuracy. From the 7,226 egos' timelines, we identified 1,054,993 retweeted tweet IDs. And then we searched these IDs in the followees' timelines. For each ego, we count the number of followees who have retweeted a post (exposures). An ego could be exposed to different posts multiple times. Therefore, we will get the information like this: an ego *i* was exposed to 200 posts only once, among which *i* retweeted 50 (probability is 50/200 = 25%); at the same time, *i* was exposed to 100 posts twice, among which *i* retweeted 50 (probability = 50%); … Finally, we calculated a sequence of probability for each ego. Fig. 6 figures were produced by averaging across different ego groups.

References

1 Gonçalves, B., Perra, N. & Vespignani, A. Modeling users' activity on twitter networks: Validation of dunbar's number. *PloS one* **6**, e22656 (2011).

2 Snijders, T. A. B. & Bosker, R. J. *Multilevel analysis: An introduction to basic and advanced multilevel modeling*. 2nd edn, (Sage, 2012).
